# Cytotoxic Potencies of Zinc Oxide Nanoforms in A549 and J774 Cells

**DOI:** 10.3390/nano14191601

**Published:** 2024-10-03

**Authors:** Nazila Nazemof, Dalibor Breznan, Yasmine Dirieh, Erica Blais, Linda J. Johnston, Azam F. Tayabali, James Gomes, Premkumari Kumarathasan

**Affiliations:** 1 Interdisciplinary School of Health Sciences, Faculty of Health Sciences, University of Ottawa, Ottawa, ON K1N 7K4, Canada; nazilanazemof@cmail.carleton.ca (N.N.); james.gomes@uottawa.ca (J.G.); 2 Environmental Health Science and Research Bureau, Healthy Environments and Consumer Safety Branch, Health Canada, Ottawa, ON K1A 0K9, Canada; dalibor.breznan@hc-sc.gc.ca (D.B.); yasmine.dirieh@hc-sc.gc.ca (Y.D.); erica.blais@hc-sc.gc.ca (E.B.); azam.tayabali@hc-sc.gc.ca (A.F.T.); 3 Metrology Research Centre, National Research Council Canada, Ottawa, ON K1A 0R6, Canada; linda.johnston@nrc-cnrc.gc.ca

**Keywords:** ZnO nanoforms, cytotoxicity, A549, J774, oxidative stress, inflammatory pathways

## Abstract

Zinc oxide nanoparticles (NPs) are used in a wide range of consumer products and in biomedical applications, resulting in an increased production of these materials with potential for exposure, thus causing human health concerns. Although there are many reports on the size-related toxicity of ZnO NPs, the toxicity of different nanoforms of this chemical, toxicity mechanisms, and potency determinants need clarification to support health risk characterization. A set of well-characterized ZnO nanoforms (e.g., uncoated ca. 30, 45, and 53 nm; coated with silicon oil, stearic acid, and (3-aminopropyl) triethoxysilane) were screened for in vitro cytotoxicity in two cell types, human lung epithelial cells (A549), and mouse monocyte/macrophage (J774) cells. ZnO (bulk) and ZnCl_2_ served as reference particles. Cytotoxicity was examined 24 h post-exposure by measuring CTB (viability), ATP (energy metabolism), and %LDH released (membrane integrity). Cellular oxidative stress (GSH-GSSG) and secreted proteins (targeted multiplex assay) were analyzed. Zinc oxide nanoform type-, dose-, and cell type-specific cytotoxic responses were seen, along with cellular oxidative stress. Cell-secreted protein profiles suggested ZnO NP exposure-related perturbations in signaling pathways relevant to inflammation/cell injury and corresponding biological processes, namely reactive oxygen species generation and apoptosis/necrosis, for some nanoforms, consistent with cellular oxidative stress and ATP status. The size, surface area, agglomeration state and metal contents of these ZnO nanoforms appeared to be physicochemical determinants of particle potencies. These findings warrant further research on high-content “OMICs” to validate and resolve toxicity pathways related to exposure to nanoforms to advance health risk-assessment efforts and to inform on safer materials.

## 1. Introduction

The enhanced use of nano-sized zinc oxide (ZnO) in applications including cosmetics, sunscreens, electronics, building materials, biosensors, and catalysts, as well as in the biomedical field [1,2,3,4], is linked to their attractive electrical, optical, magnetic, and catalytic properties in the nano-size compared to the bulk form. Also, ZnO nanoparticles (NPs) are incorporated into food packaging due to their antibacterial properties [5,6]. In addition, ZnO nanomaterials include surface-modified nanoforms to enhance their utility in various downstream applications, namely tuning optical characteristics, biocompatibility, or targeting, and to facilitate incorporation into, for instance, polymers [7]. The vast production [8] and wide range of applications of ZnO NPs can enhance the potential for human exposure and may pose health risks. Furthermore, since Zn plays an important physiological role, and its homeostasis in the biological system is tightly regulated [9], so understanding ZnO NP exposure-related biological changes is of importance in determining adverse health impacts.

Major routes of exposure to zinc oxide nanomaterials are inhalation, dermal exposure and ingestion [10,11,12]. Although there are a number of reports on the toxicity of ZnO NPs, the focus of most studies is on NP size-related effects. Furthermore, some in vitro and in vivo toxicity testing studies report on cytotoxicity, genotoxicity, and systemic toxicity of ZnO NPs [13,14,15,16,17,18,19], whereas, a few other reports [20,21] indicate no evidence of toxicity below higher exposure doses (100 µg/mL). In addition, among some in vitro toxicity testing studies conducted with the same cell type (A549 human lung epithelial cells) exposed to ZnO NPs (at 100 µg/mL), one reported moderate toxicity at this concentration [22], while other studies showed a significant reduction in cell viability only at 300 µg/mL after 18 and 24 h post exposure [23], and significant cell death at 30 and 100 µg/mL, 24 h post exposure [24]. Similarly, a slight increase in cellular oxidative stress was reported after in vitro exposure of RAW 264.7 mouse macrophage cells to ZnO NPs at relatively high dose exposures 100 µg/mL [21], while oxidative stress measured by following oxidative DNA damage in the same type of cells showed increased oxidative stress at concentrations as low as 10 µg/cm^2^ [25]. These observations suggest the need for improvement in toxicity testing for these nanomaterials. Discrepancies in toxicity findings can be attributed to the quality of these studies [26].

Nevertheless, most studies have examined size-related effects for ZnO toxicity [27], while there is paucity of research on toxicity of nanoforms (different forms of the same chemical, e.g., coated and uncoated) of ZnO. Surface modifications of ZnO NPs are not only key factors for desired technical applications but can also act as toxicity determinants [28]. In this context, it is important to gain information on toxicity characteristics associated with zinc oxide nanoforms and similarly understand the mechanistic basis of toxicity of these different nanoforms. In vitro exposure studies are relatively high throughput, less costly, not influenced by the toxicokinetic/toxicodynamic properties of these chemicals compared to in vivo exposures, and can provide mechanistic insights in combination with high-content “OMIC” analyses; thus, in vitro models are preferred for chemical toxicity testing including engineered nanomaterials, while reducing the number of animals used for this purpose. In general, manufactured NPs provide challenges in hazard identification and risk evaluation due to lack of adequate reliable physicochemical and toxicity information, thus posing difficulty for government agencies to evaluate the safety of these materials. A recent data-gap analysis identified nanoZnO as one of the engineered nanomaterials in commerce in Canada [29], and toxicity information on ZnO nanoforms is vital for assessment health risks associated with exposure to these nanomaterials.

The objective of this work was to obtain in vitro toxicity information on well-characterized ZnO nanoforms to gain insight into relative potencies and relevant toxicity mechanisms to subsequently support health risk analyses. For this purpose, human lung epithelial cells (A549) and mouse monocyte/macrophage cells (J774) were exposed to this set of ZnO nanoforms (coated and uncoated variants), and multiple cytotoxicity endpoints were measured. Furthermore, cellular oxidative stress and secreted protein responses were analyzed to gain information on related toxicity mechanisms. In addition, the relationships between the physicochemical properties of these ZnO nanoforms and the cytotoxicity of these nanoparticles were examined to identify the determinants of cytotoxic potencies.

## 2. Materials and Methods

### 2.1. ZnO Nanoforms

The ZnO nanoforms selected in this study were as follows. “Uncoated” (UC): UC-1 (30 nm, Nanostructured and Amorphous Materials Inc. NAM, Katy, TX, USA), UC-2 (35–45 nm, US Research nanomaterials Inc., USRN, Houston, TX, USA), and UC-3 (53 nm, Z-COTE, BASF, Edison, NJ, USA). Note: Sizes indicated here are those stated by the manufacturers. “Coated”: 30 nm coated with silicone oil (SO), 30 nm coated with stearic acid (SA), and 30 nm coated with (3-aminopropyl) triethoxysilane (AM) (coatings were performed by the National Research Council (NRC), Canada, from the corresponding uncoated material). All materials were obtained from NRC, Canada (Dr. L. Johnston). Also, additional reference (ZnCl_2_ (ionic form) and bulk ZnO (micron-sized, 0.11 µm [30] from Sigma Aldrich, Oakville, ON, Canada) were included in this study.

### 2.2. Physicochemical Characteristics

In this work, a chemical composition analysis was performed by Inductively Coupled Plasma Mass Spectrometry and emission spectrometry (ICP-MS and ICP-ES) on acid-digested diluted samples following methodologies reported before [30]. Transmission Electron Microscopy (TEM) was performed using the dispersed particles on formvar TEM grid (carbon film covered copper grids), and samples were imaged using FEI Technai G2 Spirit Twin TEM to measure size and shape. The Brunauer–Emmett–Teller (BET)-specific surface area was measured by nitrogen adsorption using the ASAP 2020 system from Micromeritics (Norcross, GA, USA). The hydrodynamic diameter was measured in liquid media (e.g., water or DMEM) by Dynamic Light Scattering (DLS), and surface charge was measured by Zeta potential (ZP) measurement using Zetasizer Nano ZS (Malvern Panalytical, St-Laurent, QC, Canada). A Thermo Gravimetric Analysis (TGA) was performed to assess surface group coverage using TA instruments Q500 IR (Waters Limited, Mississauga, ON, Canada). TEM, BET, TGA, DLS, amd ZP analyses were performed by the NRC laboratory (Dr. L. J. Johnston) using previously reported methods [30,31].

### 2.3. Nanoparticle Solutions for Dosing Cells

All ZnO nanoparticle stock solutions were prepared fresh for each exposure. These NP stock suspensions were prepared at 3 mg/mL in deionized water, briefly vortexed for 30 s, and sonicated in a bath sonicator (100 W, 20 kHz, VWR^®^ Ultrasonic cleaner, Mississauga, ON, Canada) for 20 min.

### 2.4. Cell Culture

J774 (mouse monocyte/macrophage) and A549 (human lung epithelial) cells from ATCC (Manassas, VA, USA) were maintained following a previously reported process [32], but with minor modifications. Cells were cultured in Dulbecco’s Modified Eagle’s medium (DMEM/High Glucose) containing phenol red with 10% fetal bovine serum (FBS) in T-75 flasks and incubated at 37 °C with 5% CO_2_. When the cells attained 80% confluency, a monolayer of cells was detached and seeded in 96-well plates at a density of 10,000 cells/well for A549 cells or 20,000 cells/well for J774 cells in 100 µL DMEM (phenol red-free (Hyclone-Cytiva, Wilmington, DE, USA)), with 10% FBS (Hyclone-Cytiva, Wilmington, DE, USA), and incubated for 24 h prior to NP exposure.

### 2.5. Exposure of Cells to ZnO NPs

After the prior 24 h incubation of cells in 96-well plates, cells were exposed to different doses (0–100 µg/cm^2^) of ZnO NPs. Briefly, for the dosing of cells with particles, NP stock suspensions (3 mg mL^–1^) were diluted with DMEM (phenol red and serum free) in water appropriately to have target doses in the range of 0–100 µg cm^–2^ (96-well surface area), sonicated for an additional 2 min, vortexed briefly, and 100 µL of the diluted NP solution was added to cells in monolayers that already contained 100 µL of DMEM containing 10% FBS. The final FBS concentration in the culture medium was 5%. Cells were incubated (37 °C, 5% CO_2_, 95% RH (relative humidity)) for 24 h prior to the analysis of cellular cytotoxicity endpoints. “No-cells” wells, containing particle suspensions only, were included in these exposure experiments to test for potential nanoparticle exposure-related interferences (e.g., optical) with the cytotoxicity endpoints analyzed in this work. Furthermore, the cells and supernatants removed were centrifuged to remove any traces of NPs prior to all cellular endpoint analyses. Exposure experiments were conducted three times (n = 3), with duplicate samples per treatment group in each exposure experiment.

### 2.6. Cytotoxicity Analysis

Cell morphology was assessed using a light microscope (40× magnification) with the Zeiss-Zen Lite microscopy software. The cytotoxicity assays employed in this work were for the measurement of cell membrane integrity (Lactate dehydrogenase (LDH) released), energy metabolism (cellular ATP), and cell viability (Cell Titer-Blue (CTB) reduction). The cytotoxicity data were normalized within an experiment for all doses (including a zero-dose control) to the grand mean value of all zero dose controls to obtain the fold effect (FE) for each particle dose.

Briefly, for the analysis of LDH released into cell supernatants, 150 µL aliquots of supernatants from NP-exposed J774 or A549 cells were collected post 24 h, transferred to a 96-well V-shape plate, and centrifuged at 2000 rpm for 10 min. Then, 50 µL of this clarified supernatant was treated with 50 µL of CytoTox-ONE assay Reagent (Promega, Madison, WI, USA). The stop solution from this assay kit was added to the reaction mixture after 10 min, and the absorbance was read at 490 nm with a POLARstar Omega spectrophotometer plate reader (BMG Lab tech, Ortenberg, Germany). Similarly, cells not treated with NPs (controls) were lysed after the 24 h post-exposure period and analyzed for total LDH content, and these measurements were used to calculate % LDH released by the cells after NP exposures.

The CTB assay was performed to assess cell viability. Briefly, 24 h post NP exposure, cell supernatants were removed and replaced with 100 µL of the fresh media containing 20% of CTB reagent (*V*/*V*) and incubated for 3 h at 37 °C. Fluorescence was measured (ƛEx = 573 and ƛEm = 600 nm) using POLARstar Omega spectrophotometer (BMG Lab tech, Ortenberg, Germany).

Cellular ATP levels were measured using the CellTiter-Glo luminescent assay kit from Promega. After 24 h of exposure to NPs, the culture media was removed from the wells, replaced with 100 µL of fresh media (5% FBS), equilibrated at room temperature (RT) for 30 min, and treated with 100 µL of CellTiter-Glo^®^ Reagent. The plate was shaken for 3 min on an orbital shaker to induce cell lysis and incubated at RT for 10 min to stabilize the luminescence signal. Supernatant was transferred to a 96-well V-bottom plate and then centrifuged for 10 min at 2000 rpm, and the luminescence signal was read using POLARstar Omega spectrophotometer plate reader (BMG Lab tech, Ortenberg, Germany).

### 2.7. Oxidative Stress Analysis

For cellular oxidative stress analysis, glutathione (GSH) and oxidized glutathione levels were measured using GSH/GSSG-Glo assay, following manufacturer’s instructions (Promega, USA, Madison). Briefly, cell supernatants were removed 24 h post NP exposures (at 30 µg/cm^2^), 50 µL of the Glutathione Lysis Reagent or Oxidized Glutathione Lysis Reagent was added to each well, and the plate was shaken at RT for 5 min on a plate shaker. Then, 50 µL of Luciferin Generation Reagent was added to each well, the plate was shaken gently and incubated at RT for another 30 min, and 100 µL of Luciferin Detection Reagent was added to each well and shaken briefly. After 15 min, the luminescence signal was measured by using a POLARstar Omega Spectrophotometer Plate Reader (BMG Lab tech, Ortenberg, Germany).

### 2.8. Multiplexed Protein Array Analysis

Secreted proteins, including cytokines, chemokines, growth factors, etc., in A549 or J774 cell supernatants were analyzed 24 h post NP exposures (at 30 µg/cm^2^) by affinity-based targeted multiplex protein array methodology using a Milliplex MAP human multiplex panel or a mouse multiplex panel, respectively, and the analysis was performed using the Bio-Plex Pro multiplex system (Bio-Rad, Hercules, CA, USA), following previously reported procedures [33]. Briefly, samples were incubated with capture antibody attached to magnetic beads, were then washed and were treated after with biotinylated detection antibody, followed by treatment with streptavidin–phycoerythrin, and then they were washed and resuspended in sheath fluid (Bio-Rad, Mississauga, ON, Canada) and analyzed using a Bioplex 200 instrument with Bioplex Manager 6.0 software for operation and data analysis (Bio-Rad, Canada). The selection of optimal multiplex panels per the different cell type was based on our previous work.

### 2.9. Endotoxin Analysis

The particle preparations mentioned above were analyzed for bacterial endotoxin using the chromogenic Limulus Amebocyte Lysate (LAL; Lonza, Walkersville, MD, USA) test, as reported previously [32].

### 2.10. Statistical and Bioinformatic Analysis

All exposure experiments were performed in triplicates. The cytotoxicity data for the NP exposure groups were normalized to the corresponding control group value (no particle treatment) to obtain the FE for each particle dose. Two-way analysis of variance was conducted with treatment and dose as factors on FE data for cytotoxicity endpoints, and data were transformed (rank-transformed) when needed to meet the conditions of normality and equal variance. Multiple comparisons testing was performed using the Holm–Sidak analysis. Also, the potency estimate (β) is derived from FE = (Dose + 1)^β^, where β represents the rate of change in dose with respect to the logarithm of fold-effect for a given endpoint [34]. The dose–response data were fitted using CurveExpert v1.4 (D. Hyams, TN, USA) to obtain these β estimates. Backward stepwise regression analysis was used to test for association between physicochemical properties of NPs and NP cytotoxic potency β values. One-way analysis of variance was performed to determine the NP treatment effects on GSH/GSSG ratio (Cellular oxidative stress status). Multiple comparisons testing was performed using Holm–Sidak analysis. All statistical tests were performed using SigmaPlot v13.0 (Systat Software, San Jose, CA, USA).

Secreted protein fold-change data (normalized to controls) were used to conduct the following bioinformatics analyses. Heat map with hierarchical clustering was employed to visualize differential patterns of protein responses as related to the different NP exposures. The analysis was performed using the hierarchical clustering option in GenePattern (https://cloud.genepattern.org/gp/pages/login.jsf, accessed on 6 June 2021) and formatted in Java TreeView (https://jtreeview.sourceforge.net; accessed on 6 June 2021). Furthermore, Ingenuity Pathway Analysis (IPA) (Ingenuity Systems, https://digitalinsights.qiagen.com/product-login/; accessed on 6 June 2021) was used to analyze for protein interactions, biofunctions and canonical pathways. Fold-change cutoff was set at 1.5, and *p* < 0.05 and z score of 2 were used for the identification of canonical pathways and disease/biological functions.

## 3. Results

### 3.1. Physicochemical Characteristics of the ZNO NPs

The chemical composition analysis of ZnO NPs contained a number of impurities in ppm quantities (Table 1 and Table 2). Some metal impurities, including Al, Ca, Cd, Fe, Mg, Mn, Na, Si, and Ti, were noticed in relatively higher quantities, especially in UC-1, UC-2, and surface-modified ZnO NPs as compared to UC-3 or the micron-sized bulk ZnO. Also, the highest level of Si was seen with SO- and AM-coated NPs, as expected, with the type of coating. Furthermore, Table 2 illustrates physicochemical properties, namely sizes provided by the manufacturer and TEM analysis results (dry state), BET surface area, DLS size for agglomeration in solution and zeta potential for surface charge, and extent of surface coating/functionalization results by TGA. The TEM data of these nanoforms showed smaller particles and larger particles, and the latter may consist of more than one particle. The BET surface area (BETSA) was greatest for the UC-2, and the DLS size was the highest for SA-coated ZnO NP. Also, transition metal contents were higher for the surface-modified NPs. All ZnO nanoforms showed positive surface charges, except for SA- and SO-coated NPs (not detectable).

### 3.2. In Vitro Cytotoxicity of Particles

**Figure 1:**
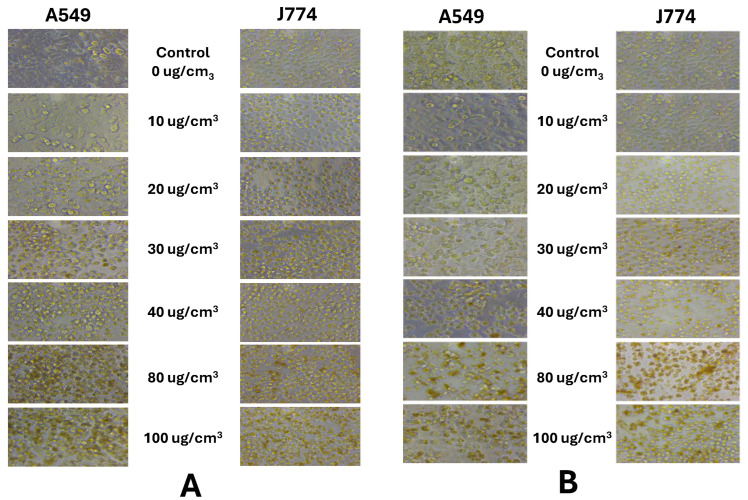
Cell morphology observed after exposure of A549 and J774 to the different doses of ZnO nanoparticles (e.g., (**A**) UC-2 and (**B**) AM): light microscopy images (40× magnification).

Representative light microscopic images (Figure 1) of both J774 and A549 cells exposed ZnO nanoforms (e.g., 30 nm ZnO NP (AM)) revealed clear dose-related changes in cell morphology. Cellular damage is seen at doses >80 µg/cm^2^ of NP exposures for both cell types.

**Figure 2:**
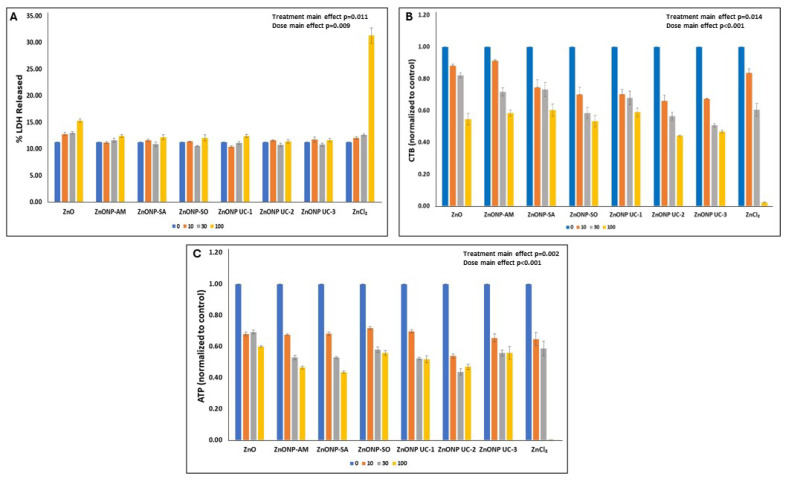
Cytotoxicity in A549 cells (mean ± SEM) after exposure (24 h) to ZnO nanoforms and the reference particles. Exposure experiments were conducted three times (n = 3), with duplicate samples per treatment group in each exposure experiment. (**A**) LDH Release, (**B**) CTB (Resazurin) Reduction, (**C**) Cellular ATP Levels.

**Figure 3:**
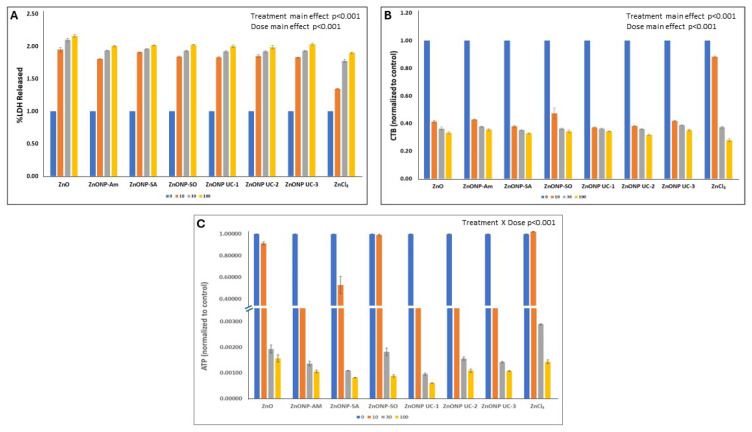
Cytotoxicity in J774 cells (mean ± SEM) after exposure (24 h) to ZnO nanoforms and reference particles. Exposure experiments were conducted three times (n = 3), with duplicate samples per treatment group in each exposure experiment. (**A**) LDH Release, (**B**) CTB (Resazurin) Reduction, (**C**) Cellular ATP Levels.

Dose-related responses of cellular cytotoxicity endpoints 24 h post NP exposures are depicted in Figure 2 and Figure 3 for the two cell types. In this work, endotoxin levels were determined to be negligible in these NPs. Cellular ATP levels and cell viability, as seen by CTB profiles, show dose-related decreases 24 h post particle exposures, while the % LDH released increased with the increasing particle dose for both A549 and J774 cell exposures. Also, for all three cytotoxicity endpoints measured in A549 cells (Figure 2), significant treatment and dose main effects (two-way ANOVA: ATP—treatment main effect (*p* = 0.002) and dose main effect (*p* < 0.001); CTB—treatment main effect (*p* = 0.014) and dose main effect (*p* < 0.001); % LDH released—treatment main effect (*p* = 0.011) and dose main effect (*p* = 0.009)) were seen with these particle exposures. In terms of particle-exposed J774 cells, % LDH released and CTB showed (Figure 3) significant treatment and dose main effects (two-way ANOVA: %LDH released—treatment main effect *p* < 0.001; dose main effect *p* < 0.001; CTB—treatment main effect (*p* = 0.031) and dose main effect (*p* < 0.001)), while cellular ATP responses displayed significant treatment X dose interaction (2-way ANOVA: *p* < 0.001). The multiple comparisons’ (Holm–Sidak) results for details on significant (*p* < 0.05) changes are provided in Supplementary Table S1.

Table 3 consists of relative particle potencies reflected by the potency estimate (β) calculated using the dose–response relationship information for each cytotoxicity assay, as described earlier [34]. Also, the relative potency ranking was performed based on average β values (β_avg_ = β_ATP_ + β_CTB_ + β_LDH_/3), where the absolute β values for the three cytotoxicity assays are used), and the results are provided in Table 3.

### 3.3. Correlation Results for Physicochemical Properties of NPs vs. Corresponding Potency Estimates

Associations between physicochemical properties of the ZnO nanoforms and potency estimates (β) for individual cytotoxicity endpoint are provided in Table 4. There are significant (*p* < 0.05) negative associations (r = −0.875) seen between BET surface area and β_ATP_ in A549 cells, as well as β_CTB_ (r = −0.905) in J774 cells. Also, the TEM size was correlated (suggestive trend) with β_CTB_ reduction in A549 cells. Furthermore, DLS size was positively (r = 0.916) correlated with β_ATP_ in J774 cells when tested for all nanoforms (n = 6); however, when tested for only the uncoated nanoforms (n = 3), this association (r = −1.000) was significantly negative (Table 4), while β_ATP_ was positively correlated with total metal (r = 0.999) and Zn (r = 1.000) contents, and a suggestive trend of negative correlation (r = −0.994) is seen between βLDH and transition metal content (Table 4).

### 3.4. Cellular Oxidative Stress

**Figure 4:**
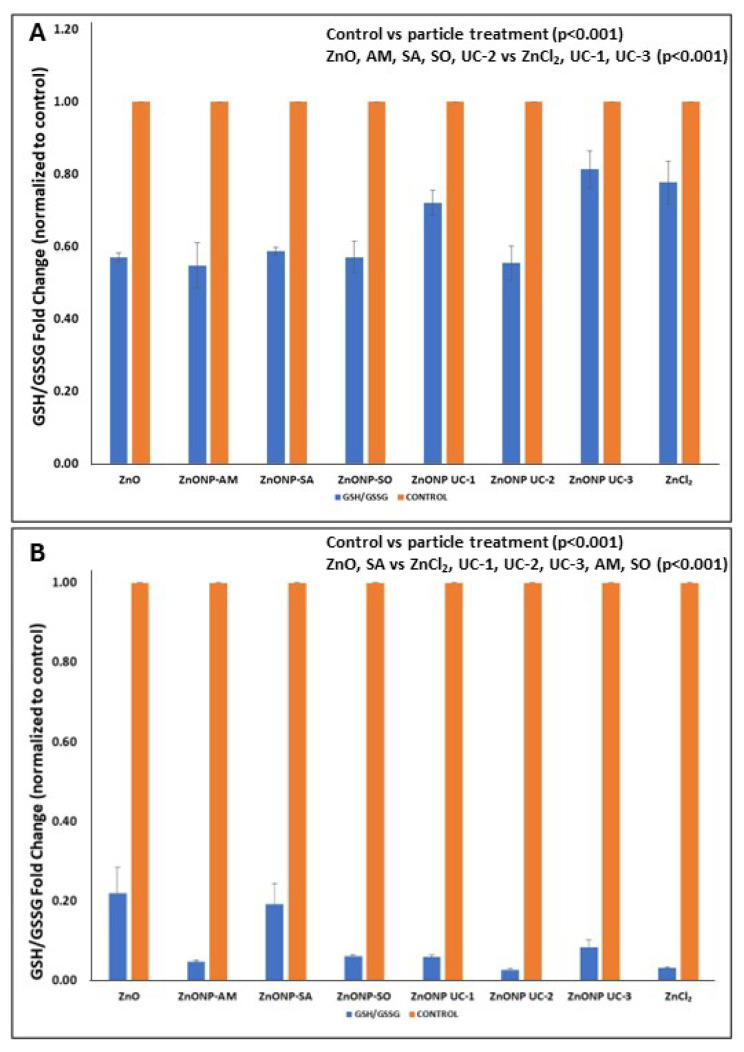
Cellular oxidative stress status in (**A**) A549 and (**B**) J774 cells after exposure to ZnO NPs, as well as to the reference particles (30 µg/cm^2^).

Furthermore, cellular oxidative stress, followed by transformation of GSH (reduced form) to GSSG (oxidized form) showed that exposures of A549 cells to ZnO nanoforms led to increased oxidative stress levels, as seen by decreased GSH/GSSG ratios notably for all surface-modified ZnO nanoforms, as well as UC-2 (Figure 4A), and this decrease was comparable, as with micron-sized zinc oxide exposures of these cells. However, the GSH/GSSG ratio decreased for all particle treatments, with ZnO NP UC-2, AM-coated, and ZnCl_2_ showing the most effect for exposed J774 cells (Figure 4B). One-way ANOVA results for the A549 cells identified that all NP treatments led to significantly decreased (*p* < 0.001) GSH/GSSG ratio (expressed as fold change -FC) values compared to the control group. Furthermore, ZnO; AM-, SA-, and SO-coated; and UC-2 NP treatment groups had significantly (*p* < 0.001) lower GSH/GSSG ratios (FC) compared to the ZnCl_2_, UC-1, UC-3 treatment groups. For the J774 cells, the one-way ANOVA results revealed that all NP treatments led to significantly decreased (*p* < 0.001) GSH/GSSG ratio (FC) values compared to the control group. Meanwhile, ZnO and SA-coated NP treatment groups exhibited a significantly (*p* < 0.001) higher GSH/GSSG ratio (FC) compared to ZnCl_2_, UC-1, UC-2, UC-3, AM-coated, and SO-coated NP treatment groups.

### 3.5. NP Exposure-Related Protein-Level Changes and Impacts on Mechanistic Pathways

**Figure 5:**
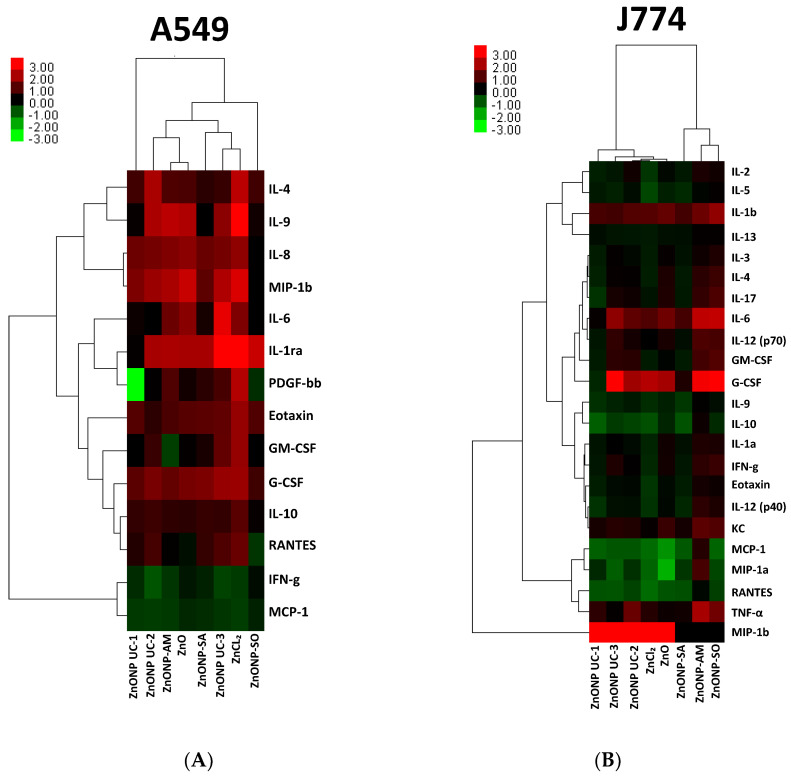
Heatmap and hierarchical clustering of secreted protein fold changes normalized to control (24 h post exposure of cells to ZnO nanoforms and reference particles: (**A**) A549 and (**B**) J774). Red—increased; green—decreased.

Heatmap and hierarchical clustering of secreted protein fold-change profiles are depicted in Figure 5A,B for ZnO nanoform-exposed A549 and J774 cells, respectively. A549 cells display many upregulated proteins with NP exposures, as with ZnCl_2_ exposure, except for SO-coated ZnO nanoforms. Meanwhile, with J774 cells, some pro-inflammatory cytokines (e.g., IL-1β, IL-6, and TNF-α) were upregulated, and anti-inflammatory IL-10 was downregulated with most ZnO nanoform exposures. Furthermore, all coated ZnO nanoform-exposure groups are seen to cluster together for J774 cells.

**Figure 6:**
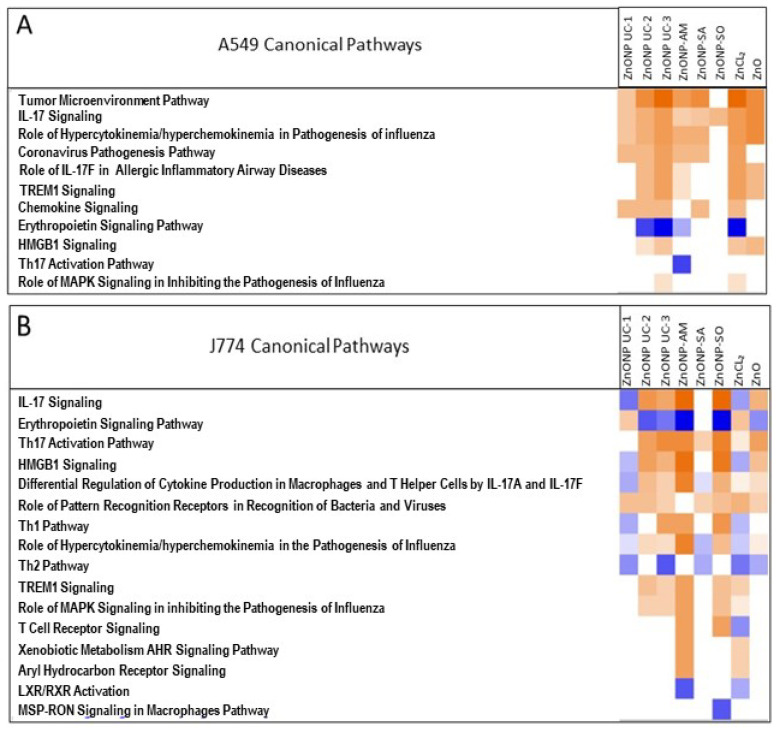
Pathway analysis results for in vitro cellular exposure (24 h) to ZnO nanoforms and the reference particles ((**A**) A549 and (**B**) J774). Orange—increased; blue—decreased.

The pathway analysis results are illustrated in Figure 6A,B. For A549 cells, inflammatory pathways, including cytokine and chemokine signaling and TREM1 and HMGB1 signaling, were upregulated. Predominant canonical pathways associated with ZnO nanoform exposures were inflammatory, cell injury, and apoptosis for J774 cells. Also, with surface coatings, notably with AM-coated NPs, the AhR (aryl hydrocarbon receptor) pathway was upregulated. The different ZnO nanoform exposures are associated with different pathway expression patterns.

## 4. Discussion

Exposure to ZnO nanoparticles has been reported to be not potent or moderately potent, or even toxic, as noted previously [17,18,20,21,35,36,37]. The inconsistency in these findings could arise due to different synthesis routes, associated contaminants, exposure conditions, physicochemical characteristics (poorly characterized), suitability of cellular assays employed (e.g., experimental artifacts), etc. These discrepancies pose a hurdle in comparing findings from the different studies. In addition, the toxicity of coated/functionalized ZnO nanoforms is poorly researched, as opposed to the uncoated pristine nanomaterial. Chemical modifications of ZnO NPs that result in different nanoforms with enhancement of specific physicochemical properties for desired downstream applications, namely an AM-coat to boost electrical properties for energy harvesting, SA-coat for superhydrophobic property for self-cleaning and oil–water separations, and SO-coat for marine antifouling surfaces [38,39,40]. These modifications can also potentially increase corresponding nanoform–cell interactions, which can be unfavorable for human health, necessitating a clear understanding of the toxicological properties of these nanoforms.

In order to overcome some of these difficulties and gain a comprehensive understanding of toxicity of various ZnO nanoforms which lack toxicity information, in this work, we have conducted in vitro exposures of a well-characterized set of uncoated and surface-modified nanoforms of ZnO (from different sources) in two different well-established cell types (human lung epithelial and mouse monocyte/macrophages) that are relevant to the pulmonary toxicity of these NPs. Even though these two functionally different cell types are from two different species, the purpose here was to gain a comprehensive understanding of the relative potencies of these nanoforms, and this approach can permit corresponding subsequent in vitro–in vivo comparisons to determine predictive capability. In addition, bulk ZnO (micron-sized) and ZnCl_2_ were also studied in parallel as reference particles. Notably, ZnCl_2_ was used as a reference to gain an understanding of soluble Zn2+ ionic form-related toxicity characteristics, while ZnO bulk material was used as a reference particle to identify nano size/form-related effects in comparison with corresponding larger-size bulk material. In addition, multiple cellular cytotoxicity assays were employed to gain information on the range of cellular effects. Also, cell-free NP exposures were conducted to correct for any potential experimental artifacts or NP-related optical interferences in these assays.

Physicochemical property data suggested some consistencies in TEM particle sizes and the manufacturer’s information. In addition, taken together, the relative BETSA and DLS values for these nanoforms also are consistent with the sizes of these NPs, except for the larger DLS value for the ZnONP SA nanoform, which potentially can be attributed to the type of surface coating. Corresponding DLS values and solubility values in DMEM (cell culture medium) for some of these ZnO nanoforms used in this study have been reported previously [41,42,43]. Also, chemical analysis data show a number of contaminant reactive metals, including transition metals (Table 1 and Table 2).

Exposure to high doses of ZnO nanoforms (80–100 µg/cm^2^) resulted in cell damage or disintegration (Figure 1). Findings on cellular cytotoxicity identified dose-related ZnO nanoform-specific adverse effects in terms of cell viability (CTB), cellular metabolic status (ATP), and some effects on cell membrane integrity (%LDH released) in human lung epithelial A549 cells. Endotoxin levels were negligible in these NPs. Meanwhile, this work also showed that cell membrane permeability, cellular ATP, and cell viability were affected dramatically in the phagocytic monocyte/macrophage J774 cells, as compared to A549 lung epithelial cells, suggesting differences in NP–cell-type interactions. These cytotoxicity findings for the two cell types shed some light on the behavior of these cell types upon exposure to the contrasting reference particles, the bulk ZnO (micro particles) and the soluble ionic ZnCl_2_, where A549 cells were relatively more responsive to ZnCl_2_ (ionic Zn^2+^) compared to that of the micron-sized ZnO, whereas the micron-sized bulk ZnO exposure led to a relatively heightened cytotoxicity response in phagocytic J774 cells compared to that of ZnCl_2_. These observations on the reference particle exposures suggest differences in cellular uptake of the less soluble and highly soluble particles. Uptake of molecules/particles by the epithelial (A549) cells is by pinocytosis (perhaps constitutive or partly receptor mediated) mechanisms [44,45]. Although relatively smaller particles can be taken up by monocyte/macrophage (J774) cells by endocytosis mediated by clathrin- and caveolae-dependent mechanisms, larger particles are reported to be taken up by F-actin dependent phagocytosis mechanism [46]. These cell type-specific particle-uptake mechanisms with potential influence of solubility of particles [47] can aid in our understanding of the internal localization of different particles and, thus, relative potencies.

Moreover, CTB and ATP levels decreased relatively more compared to the micron-sized bulk ZnO for most of the nanoform exposures at low doses, especially for the two uncoated UC-2 and UC-3 ZnO nanoform-exposed A549 cells, as with ZnCl_2_, but not at the highest dose of exposure. This finding may be suggestive of a Zn^2+^ ion release from these nanoforms and, perhaps, an associated uptake mechanism by this cell type. The low dose exposure-related cellular responses to the ZnO nanoforms can be attributed to the increased solubility of these nanoparticles at low doses, as reported previously [43], where relatively high solubility of UC-2 was seen at 10 µg/mL in DMEM, whereas, at high doses (100 µg/mL) of exposure, the solubility was lower. Thus, at high dose exposures of ZnO nanoforms, cellular responses can be explained, in addition to solubility, by other physicochemical characteristics of these particles, including size, surface area, surface groups, agglomeration state, and surface charge [48,49,50]). In contrast, J774 cells exposed to ZnO nanoforms exhibited increased LDH, exhibiting effects on membrane permeability, as well as dose-related decreases in CTB and ATP levels, but similar behavior was seen with the bulk ZnO, and ZnCl_2_ exhibited somewhat similar responses in this cell type. Of the cytotoxicity responses examined, ATP levels in J774 cells showed contrasts in responses for the nanoforms, for the bulk form, and for ZnCl_2_ which can be attributed to the cellular uptake of particles by phagocytosis. Also, all ZnO nanoform exposure-related cellular responses were more pronounced in J774 cells as compared to that of A549 cells, and this observation is in line with previous reports [51]. Differences in the cellular uptake of ZnO nanoparticles by epithelial cells (bronchial) and by phagocytic cells have been shown previously [52]. Although, with other cytotoxicity endpoints, significant (*p* < 0.05) treatment and dose main effects were seen, cellular ATP levels in particle-exposed J774 cells displayed treatment X dose interactions (Supplementary Table S1). The average cytotoxic potency estimate (β_avg_)-based potency ranking for these ZnO nanoforms for both cell types, A549 and J774 cells, also showed that this rank order was cell type-dependent (Table 3).

The backward stepwise regression analysis revealed that some of the physicochemical properties of the NPs influenced the cytotoxic responses (Table 4). Notably, the TEM size; BET surface area (BETSA); DLS size, suggesting the agglomeration state; and total and transition metal contents were some of the physicochemical properties that appeared to influence these NP potencies, based on the cell type. The increased metal impurities in some of the ZnO nanoforms (e.g., Cd, Fe, and Mn) that can influence cytotoxicity responses are provided in Table 1. It is also interesting to note that the highest level of Ce was measured in UC-2 (Table 1). UC-2 is associated with a relative potency rank order of 2 in both cell types (Table 3). UC-3 also was shown to have the next higher level of Ce compared to the other nanoforms and ranked third in terms of relative potency in both cell types. This finding is consistent with a previous report on CeO_2_ enhancing the toxicity of ZnO NPs in lung epithelial cells [53].

ZnO nanoform exposures in this work showed cell type-specific cellular oxidative stress. For instance, with the A549 lung epithelial cells, the GSH/GSSG ratios were higher for the ZnCl_2_, UC-1, and UC-3 treatments compared to the other particle exposures. Meanwhile, with J774 cells, these ratios (FC) were higher for the ZnO and SO-coated groups compared to other particle exposures. Oxidative-stress responses were higher in J774 cells compared to A549 cells, as seen from the GSH-to-GSSG conversion ratios: the lower the GSH/GSSG values, the higher the cellular oxidative stress levels. These results are in line with previous reports on ZnO nanoparticle exposure-related oxidative stress and adverse biological consequences [14,37,54,55,56,57]. Interestingly, Zn from inhaled ambient air particles has been implicated in cellular oxidative stress and downstream cardiovascular effects and is considered a determinant of cardiovascular toxicity of ambient air particles [58,59]. Here, the reference ionic material ZnCl_2_ also exhibited a cell type-specific increase in oxidative stress notably; the highest GSH oxidation was seen in J774 cells for this particle compared to the other treatments, whereas in A549 cells, ZnCl2 showed relatively decreased oxidation of GSH compared to ZnO nanoparticles, which may be attributed to cellular uptake of the different states of Zn by the different cell types (Figure 4A,B) and impact on molecular targets, namely proteins. Zn^2+^ ion is a constituent of various key protein structures, namely the antioxidant enzyme cytosolic superoxide dismutase (CuZnSOD) and the extracellular matrix modifiers, the matrix metalloproteinases (MMPs). It is interesting to note the switch in relative oxidative stress levels associated with the reference particles (micron-sized bulk ZnO and ZnCl_2_) between the two cell types.

The analysis of the targeted secreted proteins after in vitro exposure of both A549 and J774 cells to ZnO nanoforms (at the low dose 30 μg/mL) exhibited cell-specific and nanoform-specific inflammatory protein changes (Figure 5), and these observations are in line with the report on acute-phase inflammatory response after pulmonary exposure to low doses of ZnO nanoparticles [60]. Also, it was interesting to note that, in A549 cells, there was a differential increase in pro-inflammatory cytokines (e.g., IL-6, IL-8, and IL-1ra), with no or relatively small increases in anti-inflammatory IL-10 levels, after exposure to most of the ZnO nanoforms tested in this study. Meanwhile, with J774 cells, increased differential expression of pro-inflammatory cytokines (e.g., IL-6, MIP-1β, G-CSF, and TNF-α) was seen for the different ZnO nanoforms, but the anti-inflammatory IL-10 level was decreased across all particle exposures, with the exception of ZnO-AM and bulk ZnO, suggesting differential pro-inflammatory response after exposure to these ZnO nanoform exposures in these cells. These findings are consistent with some previous in vitro exposure studies conducted with ZnO NPs in similar cells [56,61,62,63] and also with inhalation exposure studies [35,64]. In addition, similar proinflammatory responses are known to be a consequence of enhanced oxidative stress [52], such as the tier-two response that drives proinflammatory status through JNK activation that can lead to an increase in IL-8 in BEAS-2B epithelial cells and TNF-α levels in RAW 264.7 cells, and this is in line with our observations on increased IL-8 in A549 epithelial cells and TNF-α levels in J774 cells. There are a few reports on proteomic analyses performed to study the molecular-level changes after ZnO exposures; however, the exposed cell types and conditions are different [65,66]. Moreover, proteomic analysis of bronchoalveolar lavage of rats exposed to ZnO NPs by LC-mass spectrometry showed activation of mainly pathways of immune and inflammatory processes [35], a result which is in line with our current findings.

Pathway analysis results based on limited targeted secreted protein expressions also identified increased inflammatory signaling in J774 and A549 cells for most ZnO nanoforms. For instance, IL-17, TREM1, and HMGB1 (high mobility group box 1) signaling were seen to be upregulated in both cells for UC-2 and UC-3 nanoforms of ZnO. TREM1 overexpression is known to cause severe inflammation in A549 cells [67]. In addition, cellular reactive oxygen species (ROS) are known to lead to HMGB1 secretion and are associated with various cell-death modes (apoptosis, necrosis, necroptosis, and ferroptosis), and HMGB1 signaling is implicated in leading to proinflammatory status [68]; this is consistent with ROS profiles, as well as inflammatory pathway upregulation (IL17 and TREM1), for both cell types after UC-2 ZnO nanoform exposure. Meanwhile, for J774 cells, consistency between ROS production and inflammatory pathway upregulation was noticed for UC-2, UC-3, and AM-, SO-coated nanoforms. These responses were also in line with cellular ATP profiles after exposure to these ZnO nanoforms, suggesting potential cell injury and cell death processes, as mentioned earlier. Furthermore, some ZnO nanoform exposures were associated with the downregulation of the erythropoietin signaling pathway. Erythropoietin is known to inhibit lung epithelial cell apoptosis [69]; thus, the downregulation of this signaling pathway after ZnO NP exposures suggests a pro-apoptotic state, and it is in line with the inverse relationship with HMGB1 signaling expressions (Figure 6), as noted above, for these cell types, for the specific ZnO nanoforms. UC-2 and UC-3 ZnO nanoforms showed similar pathway profiles in both cell types, and with J774 cells, AM- and SO-coated ZnO nanoforms also exhibited somewhat similar pathway profiles. Also, in J774 cells, it was interesting to note that the AM-coated ZnO nanoform exposure showed activation of the xenobiotic metabolism AhR signaling pathway. This is in line with a previous report on gold nanoparticles coated with an AhR ligand that activated this pathway in dendritic cells [70]. The biological processes associated with these perturbed pathways after ZnO nanoform exposures included the production of reactive oxygen species, apoptosis, and necrosis.

Collectively, the findings from this work suggest the importance of the use of multiple cell lines and multiple assays, along with the use of contrasting types of reference particles, in gauging the cytotoxicity behavior of these ZnO nanoforms. Although the solubility of ZnO nanoforms and release of Zn^2+^ ions can contribute partly to the toxicity of these particles, perhaps mediating oxidative stress, chemical contaminants present in these particles also can contribute to oxidative stress. For instance, increased oxidative stress after exposure to UC-2 ZnO nanoform in both cell types compared to the other ZnO NPs can perhaps be attributed to the presence of relatively higher levels of Ce as a chemical contaminant in this NP, in line with a previous report on the combination of Ce and ZnO NP exposure-related induction of oxidative stress-mediated inflammation in rats [71]. Similarly, physicochemical characteristics size, surface area, surface groups, and agglomeration can contribute to cellular cytotoxicity, in addition to solubility, of NP isoforms by influencing NP–cell interactions and, thus, the uptake of NPs by cells. Furthermore, the findings on targeted cell-secreted protein markers provided some new insights into ZnO nanoform exposure- and cell type-related mechanistic pathways and biological processes consistent with these NP exposure-linked cellular oxidative stresses consistent with the observed cellular cytotoxicity effects, notably the fate of cellular ATP levels/cytotoxicity and cell injury/death. It will be useful to incorporate advanced in vitro models, such as co-culture models, organoids, or an air–liquid interface ALI model, to physiologically engage more relevant culture conditions in the toxicity testing of nanoforms and to compare findings from the 2D cell culture-based experiments in future work in order to enhance predictive capability.

## 5. Conclusions

Our findings from this work identified cell type- and nanoform-specific cytotoxicity responses after exposure to these ZnO nanoparticles. Size, surface area, agglomeration state, and contaminant metal content appear to influence cell-specific ZnO nanoparticle uptake and thus can be determinants of ZnO nanoform exposure-related cell type-specific cytotoxicity. Also, ZnO nanoform exposure-led elevation in cellular oxidative stress and related upregulated inflammatory signaling pathways, notably after exposure to UC-2, UC-3, Am-coated, and SO-coated nanoforms and are in line with two important key events known to occur with ZnO nanoparticle exposures that lie on the path to cell injury. These findings can support the read-across strategy in the health risk assessment of these nanoforms. Future research is required to expand our current understanding of cellular toxicity pathways using high-content OMIC analyses to refine/validate existing adverse outcome pathway models to realize nanoform-specific in-depth toxicity mechanisms.

## Figures and Tables

**Table 1:** Elemental analyses results (ICP-ES and ICP-MS) for the different particles.

Elements (ppm)	ZnO	ZnONP AM	ZnONP SA	ZnONP SO	ZnONP UC-1	ZnONP UC-2	ZnONP UC-3	ZnCl₂
As	0.01	0.04	0.02	<0.01	0.01	0.09	0.03	<0.01
Al	2.15	75.47	104.51	41.28	79.88	50.67	20.05	1.20
Ba	0.27	3.60	1.95	1.22	2.28	14.67	0.29	0.06
Bi	0.07	0.02	0.02	0.01	<0.01	0.03	0.01	0.23
Ca	17.92	347.11	876.69	1067.04	641.47	1835.73	20.43	16.67
*Cd*	11.86	0.42	0.71	0.12	1.09	0.62	3.44	2.22
Ce	0.02	0.03	0.02	0.01	0.01	1.28	1.11	0.41
*Co*	<0.01	0.15	0.09	0.04	0.11	0.26	<0.01	<0.01
*Cr*	0.36	0.46	0.46	0.23	0.44	4.21	0.10	0.33
Cs	<0.01	<0.01	<0.01	0.00	0.00	<0.01	0.02	0.04
*Cu*	0.75	3.24	5.24	2.50	3.89	1.63	10.39	0.38
Dy	<0.01	<0.01	<0.01	<0.01	<0.01	0.04	<0.01	<0.01
Er	<0.01	<0.01	<0.01	<0.01	<0.01	0.02	<0.01	<0.01
Eu	<0.01	<0.01	<0.01	<0.01	<0.01	0.01	<0.01	<0.01
*Fe*	3.35	287.07	19.36	22.20	18.24	167.89	10.18	2.90
Gd	<0.01	<0.01	<0.01	<0.01	<0.01	0.12	0.01	<0.01
*Hf*	<0.01	0.01	0.02	<0.01	0.01	<0.01	<0.01	<0.01
Ho	<0.01	<0.01	<0.01	<0.01	<0.01	0.01	<0.01	<0.01
K	8.19	14.44	13.80	18.56	11.25	20.21	10.59	7.02
*La*	<0.01	0.01	0.02	<0.01	0.01	10.67	0.02	0.02
Lu	<0.01	<0.01	<0.01	<0.01	<0.01	<0.01	<0.01	<0.01
Mg	1.21	112.06	173.98	220.07	119.84	538.70	2.47	1.01
*Mn*	0.17	1.54	4.40	3.65	1.01	64.47	0.13	0.09
*Mo*	0.02	<0.01	<0.01	<0.01	0.02	0.07	0.01	0.02
Na	7.94	780.06	363.74	1724.20	949.48	217.37	11.68	3.74
*Nb*	<0.01	<0.01	0.01	0.01	0.02	<0.01	<0.01	<0.01
Nd	<0.01	0.01	<0.01	<0.01	<0.01	1.20	<0.01	0.01
*Ni*	0.07	0.63	1.24	0.59	0.60	21.68	0.09	0.19
Pr	<0.01	<0.01	<0.01	<0.01	<0.01	0.42	<0.01	<0.01
Rb	0.02	0.02	0.02	0.02	0.02	0.04	0.02	0.02
Sb	0.01	0.01	0.08	0.01	0.03	0.03	0.01	0.01
*Sc*	<0.01	0.03	<0.01	0.06	0.01	<0.01	<0.01	<0.01
Si	10.46	1173.68	46.21	2718.81	24.77	36.37	27.72	9.04
Sm	<0.01	<0.01	<0.01	<0.01	<0.01	0.05	<0.01	<0.01
Sr	0.02	1.80	5.33	4.32	3.50	13.56	0.02	0.06
*Ta*	<0.01	<0.01	<0.01	<0.01	<0.01	<0.01	<0.01	<0.01
Tb	<0.01	<0.01	<0.01	<0.01	<0.01	0.01	<0.01	<0.01
Th	<0.01	0.02	0.05	0.01	0.02	<0.01	0.01	<0.01
*Ti*	0.97	320.69	382.85	489.93	228.36	1.54	1.32	0.66
Tm	<0.01	<0.01	<0.01	<0.01	<0.01	<0.01	<0.01	<0.01
U	<0.01	<0.01	<0.01	<0.01	<0.01	0.13	<0.01	<0.01
*V*	0.01	0.05	0.04	0.02	0.02	0.07	0.03	0.02
Yb	<0.01	<0.01	<0.01	<0.01	<0.01	0.01	<0.01	<0.01
*Zn*	770,268	763,278	715,683	721,911	760,799	718,568	753,952	616,834
*Zr*	0.05	0.30	0.91	0.12	0.47	0.06	0.02	0.02

Elemental composition data was determined using the ICP-MS/AES analysis of the particles; Data are presented in ppm (mg element/kg particles); Transition metals are highlighted in italic.

**Table 2:** Physicochemical properties of ZnO nanoforms.

Nano Particle	TEM (nm)	BETSA (m^2^/g)	DLS (nm)	PDI	ZP (mV)	Transition Metals (ppm)^a^	TGA µmol/g (%)
ZnONP AM	30 (20,200)	16.3	212	0.12	19.3	613.8	119 (0.69%)
ZnONP SA	10–30(20, 50–100)	29	420	0.13	nd	413. 7	165 (4.69%)
ZnONP SO	30 (15, 100)	14.1	na	na	nd	519.1	(4.21%)
ZnONP UC-1	30 (<100)	21.6	223	0.19	23.2	252.7	
ZnONP UC-2	35–45 (20–40)	34.5	278	0.39	23	261.8	
ZnONP UC-3	53 ± 23 (20–30, >100)	11.9	232	0.14	30.5	22.2	

Manufacturer’s information on size of particle: TEM—Transmission electron microscopy of NPs in dry state (TEM analysis results are in paranthesis & small and large size particles were observed by TEM. Note: the higher size may comprise of more than one particle). BETSA—BET surface area; DLS—Dynamic light scattering sizes (in solution); ZP—Zeta potential (surface charge); TGA—Thermogravimetric analysis for extent of coating/functional groups on NPs. ^a^ Chemical composition analysis by ICP-ES/ICP-MS.

**Table 3:** Relative particle potencies and rank orders for the different cell types.

Nano Particle	A549					J774				
	ATP (β)	LDH (β)	CTB (β)	Consensus Average β	Rank	ATP (β)	LDH (β)	CTB (β)	Consensus Average β	Rank
ZnO	0.120	0.058	0.089	0.089	8	0.348	0.189	0.295	0.277	7
ZnONP AM	0.173	0.014	0.093	0.093	7	1.600	0.170	0.280	0.683	1
ZnONP SA	0.177	0.008	0.106	0.097	4	0.493	0.174	0.311	0.326	6
ZnONP SO	0.140	0.003	0.146	0.096	5	0.328	0.172	0.277	0.259	8
ZnONP UC-1	0.160	0.007	0.121	0.096	6	1.233	0.170	0.305	0.569	4
ZnONP UC-2	0.213	0.001	0.173	0.129	2	1.381	0.170	0.310	0.620	2
ZnONP UC-3	0.152	0.003	0.176	0.110	3	1.258	0.172	0.282	0.571	3
ZnCl_2_	0.265	0.172	0.220	0.219	1	1.258	0.145	0.205	0.536	5

β—potency estimate

**Table 4:** Backward stepwise regression results for associations between cytotoxic potency estimate (β) vs. physicochemical properties of ZnO nanoforms.

Cell Type	Predictor(s)
A549	β_ATP_: BETSA (*p* = 0.023)
	β_LDH_: TEM (*p* = 0.049), BETSA (*p* = 0.046)
	β_CTB_: TEM (*p* = 0.039), Total Metals (*p* = 0.032)
J774	β_ATP_: DLS (*p* = 0.029)
	β_LDH_: BETSA (*p* = 0.054), DLS (*p* = 0.028)
	β_CTB_: BESTA (*p* = 0.041)

## Data Availability

Data is contained within the article.

## Supplementary Materials

The following supporting information can be downloaded at https://www.mdpi.com/article/10.3390/nano14191601/s1, Table S1: 2-Way ANOVA: Multiple comparisons (Holm-Sidak) Results.
