# Supplementary material for: Cytotoxic Potencies of Zinc Oxide Nanoforms in A549 and J774 Cells
Source: Nanomaterials (Basel). 2024 Oct 3;14(19):1601. doi: 10.3390/nano14191601 (PMC11482475; doi:10.3390/nano14191601)
Supplement: Supplementary file 1 [file nanomaterials-14-01601-s001.zip › nanomaterials-3150424-supplementary.pdf]

Supplementary Table 1: 2-Way ANOVA: Multiple comparisons (Holm-Sidak) Results

A549 (CTB)

Treatment	p<0.05
None Specified	
**Dose**	**p<0.05**
0 vs 10	Yes
0 vs 30	Yes
0 vs 100	Yes
10 vs 30	Yes
10 vs 100	Yes
30 vs 100	Yes

A549 (LDH)

Treatment	p<0.05
None Specified	
**Dose**	**p<0.05**
0 vs 100	Yes
30 vs 100	Yes

A549 (ATP)

Treatment	p<0.05
ZnO vs ZnONP UC-2	Yes
ZnO vs $ZnCl_2$	Yes
ZnONP UC-3 vs ZnONP UC-2	Yes
**Dose**	**p<0.05**
0 vs 10	Yes
0 vs 30	Yes
0 vs 100	Yes
10 vs 30	Yes
10 vs 100	Yes
30 vs 100	Yes

J774 (CTB)

Treatment	p<0.05
None Specified	
**Dose**	**p<0.05**
0 vs 10	Yes
0 vs 30	Yes
0 vs 100	Yes
10 vs 30	Yes
10 vs 100	Yes
30 vs 100	Yes

J774 (LDH)

Treatment	p<0.05
ZnO vs $ZnCl_2$	Yes
ZnONP-SA vs $ZnCl_2$	Yes
ZnO vs ZnONP UC-1	Yes
ZnONP-SO vs $ZnCl_2$	Yes
ZnO vs ZnONP-AM	Yes
ZnO vs ZnONP UC-2	Yes
ZnONP UC-3 vs $ZnCl_2$	Yes
ZnO vs ZnONP UC-3	Yes
ZnONP UC-2 vs $ZnCl_2$	Yes
ZnONP-AM vs $ZnCl_2$	Yes
ZnO vs ZnONP-SO	Yes
ZnONP UC-1 vs $ZnCl_2$	Yes
**Dose**	**p<0.05**
0 vs 10	Yes
0 vs30	Yes
0 vs 100	Yes
10 vs 30	Yes
10 vs 100	Yes
30 vs 100	Yes

J774 (ATP)

Treatment (within Dose 10)	p<0.05
$ZnCl_2$ vs ZnONP-AM	Yes
$ZnCl_2$ vs ZnONP UC-1	Yes
$ZnCl_2$ vs ZnONP UC-2	Yes
$ZnCl_2$ vs ZnONP UC-3	Yes
$ZnCl_2$ vs ZnONP-SA	Yes
$ZnCl_2$ vs ZnO	Yes
ZnONP-SO vs ZnONP UC-1	Yes
ZnONP-SO vs ZnONP UC-2	Yes
ZnONP-SO vs ZnONP UC-3	Yes
ZnONP-SO vs ZnONP-SA	Yes
ZnONP-SO vs ZnONP-AM	Yes
**Treatment (within Dose 30)**	**p<0.05**
$ZnCl_2$ vs ZnONP UC-1	Yes
$ZnCl_2$ vs ZnONP-SA	Yes
**Treatment (within Dose 100)**	**p<0.05**
$ZnCl_2$ vs ZnONP UC-1	Yes
ZnO vs ZnONP UC-1	Yes
$ZnCl_2$ vs ZnONP-SA	Yes
**Dose (within treament ZnO)**	**p<0.05**
0 vs 10	Yes
0 vs 30	Yes
0 vs 100	Yes
10 vs 30	Yes
10 vs 100	Yes
**Dose (within treament ZnONP-AM)**	**p<0.05**
0 vs 10	Yes
0 vs 30	Yes
0 vs 100	Yes
10 vs 30	Yes
10 vs 100	Yes
**Dose (within treament ZnONP-SA)**	**p<0.05**
0 vs 10	Yes
0 vs 30	Yes
0 vs 100	Yes
10 vs 30	Yes
10 vs 100	Yes
30 vs 100	Yes
**Dose (within treament ZnONP-SO)**	**p<0.05**
0 vs 30	Yes
0 vs 100	Yes
10 vs 30	Yes
10 vs 100	Yes
30 vs 100	Yes
**Dose (within treament ZnONP UC-1)**	**p<0.05**
0 vs 10	Yes
0 vs 30	Yes
0 vs 100	Yes
10 vs 30	Yes
10 vs 100	Yes
**Dose (within treament ZnONP-UC-2)**	**p<0.05**
0 vs 10	Yes
0 vs 30	Yes
0 vs 100	Yes
10 vs 30	Yes
10 vs 100	Yes
30 vs 100	Yes
**Dose (within treament ZnONP-UC-3)**	**p<0.05**
0 vs 10	Yes
0 vs 30	Yes
0 vs 100	Yes
10 vs 30	Yes
10 vs 100	Yes
**Dose (within treament $ZnCl_2$)**	**p<0.05**
0 vs 10	Yes
0 vs 30	Yes
0 vs 100	Yes
10 vs 30	Yes
10 vs 100	Yes
30 vs 100	Yes
